# Supplementary material for: Comparative multiomics analysis of cell physiological state after culture in a basket bioreactor
Source: Sci Rep. 2022 Nov 23;12:20161. doi: 10.1038/s41598-022-24687-4 (PMC9686226; doi:10.1038/s41598-022-24687-4)
Supplement: Supplementary file 1 — Supplementary Information 1. [file 41598_2022_24687_MOESM1_ESM.zip › raw data/Proteomic raw data/2.func.anno/2.1.GO/GO.anno.pdf]

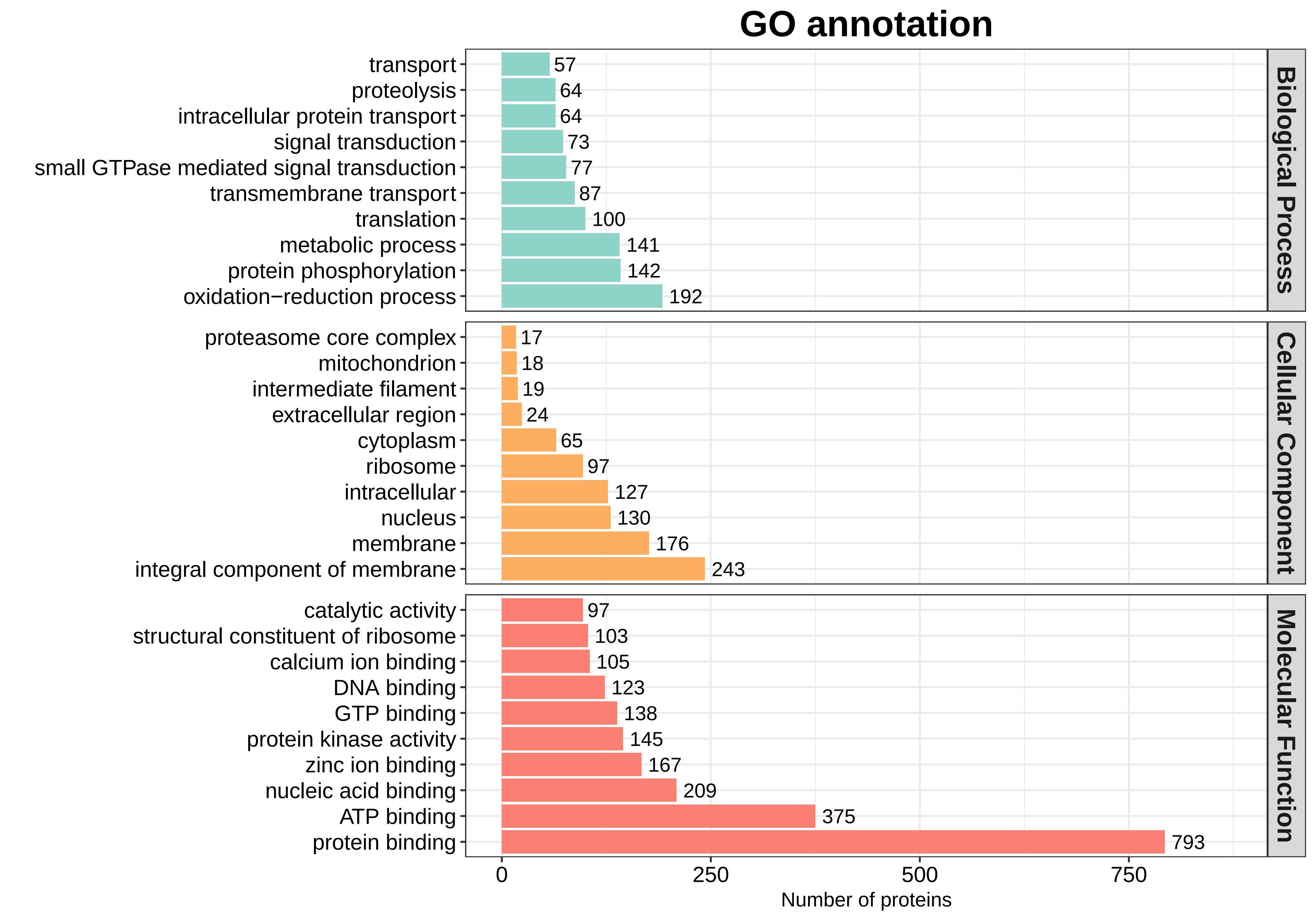
GO annotation
transport 57
proteolysis 64
intracellular protein transport 64
signal transduction 73
small GTPase mediated signal transduction 77
transmembrane transport 87
translation 100
metabolic process 141
protein phosphorylation 142
oxidation−reduction process 192
Biological Process
proteasome core complex 17
mitochondrion 18
intermediate filament 19
extracellular region 24
cytoplasm 65
ribosome 97
intracellular 127
nucleus 130
membrane 176
integral component of membrane 243
Cellular Component
catalytic activity 97
structural constituent of ribosome 103
calcium ion binding 105
DNA binding 123
GTP binding 138
protein kinase activity 145
zinc ion binding 167
nucleic acid binding 209
ATP binding 375
protein binding 793
Molecular Function
0
250
500
750
Number of proteins
